# Construction and differential analysis of testicular atlas between 10-week-old and 23-week-old ducks using single-cell RNA sequencing

**DOI:** 10.1016/j.psj.2025.105715

**Published:** 2025-08-22

**Authors:** Zhiyun Tao, Wenjuan Xu, Weitao Song, Lizhi Lu, Shuangjie Zhang, Hongxiang Liu, Zhicheng Wang, Haotian Gu, Chunhong Zhu, Huifang Li

**Affiliations:** a Department of waterfowl breeding and production, Jiangsu Institute of Poultry Sciences, Yangzhou 225125, China; b institute of Livestock and veterinary Research, Zhejiang Academy of Agricultural Sciences, Hangzhou 310021, China

**Keywords:** Duck, Testes, Single cell RNA-sequencing, Marker gene, Comparative transcriptomic

## Abstract

While spermatogenesis has been extensively characterized in mammals, its molecular underpinnings in avian species remain largely unexplored. To address this knowledge gap, we performed single-cell transcriptomic profiling of duck testes across developmental stages (10-week immature vs. 23-week mature). Our analysis generated a comprehensive cellular atlas comprising 54,702 cells, resolving eight germ cell clusters (three spermatogonia [SPG], three spermatocytes [SPC], two spermatozoa [SPT]) and nine somatic populations, including peritubular myoid cells, immune subsets (T cells, macrophages, granulocytes), endothelial cells, Leydig cells, and three Sertoli cell subtypes, each defined by unique marker gene signatures. Furthermore, novel marker genes were identified, including EXFABP for granulocyte, ARHGAP15 for T cell regulation, FDX1 specific to Leydig cells (LC), and TSSK3/TSSK2 linked to elongated spermatid formation (SPT). Notably, we identified some novel molecular markers distinguishing these populations. Pseudotemporal trajectory reconstruction of germline development revealed stage-specific enrichment of ribosome, endoplasmic reticulum protein processing, and autophagy pathways. Core regulators *MRPL13, MRPL2, MRPL22, MRPS14, MRPS7* (ribosome), *HSPA5* (ER stress response), and *PIK3C3* (autophagy) emerged as molecular hubs showing progressive downregulation during differentiation. Comparative transcriptomic analysis of germ cells and Sertoli cells between immature (IMT) and mature (MT) testes revealed significant enrichment of the spliceosome pathway in both germ and Sertoli cells. Critical spliceosome components *SNRPG, SF3B3*, and *SNRPF* exhibited coordinated downregulation during testicular maturation, suggesting their role as negative regulators of spermatogenic progression. This study establishes the first high-resolution cellular blueprint of avian spermatogenesis, delineating regulatory networks of duck testis cell development. Our findings provide valuable datasets and mechanistic insights into the evolutionary specialization of reproductive strategies in poultry.

## Introduction

Spermatogenesis, as the process by which spermatozoa are formed from immature germ cells in male individuals, is a focus of reproductive research. Reproductive cells gradually proliferate and differentiate from undifferentiated spermatogonia (SPG) (also called spermatogonial stem cells (SSCs)) to spermatocytes (SPC), which undergo meiosis to form haploid sperm cells (SPT), ultimately becoming sperm. Meanwhile, germ cell differentiation also requires specialized somatic support, including from Sertoli cells (SC), Leydig cells (LC), peritubular myoid cells (PMC), and muscle cells that surround the seminiferous tubules (Sohni et al., 2019). During the process of spermatogenesis, however, the heterogeneity, gene expression patterns, and temporal differentiation trajectories of germ cells and somatic cells differ among species, and little is known about the process of spermatogenesis in poultry.

Rapid developments in single-cell sequencing have allowed its application in various fields, such as tumors, microbiology, neurology, and reproductive biology. Single-cell sequencing can be used to sequence and quantify the whole genome of germ cells at the single-cell level, thus helping us to understand their occurrence and development. The technique has accordingly been widely used in the study of mammalian embryos and reproductive organs (Shami et al., 2020), and numerous studies have examined the process of spermatogenesis in mammals, including in mouse testes, where four germ cell clusters were defined (Green et al., 2018). In addition, six types of somatic cells and five subtypes of spermatogenic cells were identified in dairy goats and the specific marker genes *TKTL1* and *AES* were screened and identified for spermatogonia (Xiu et al., 2021). In humans, testicular cells are divided into eight germ and five somatic cell clusters (Guo et al., 2018), and specific markers of undifferentiated human SPG (*ID4* and *FGFR3*) have been identified (Guo et al., 2017; Sachs et al., 2014). Recent studies have revealed the preservation and differential characteristics of molecular markers and cellular states of spermatogenesis during evolution from mice to humans (Shami et al., 2020). These datasets provide a rich foundation for future targeted mechanism research into primate germ cell development and *in vitro* gamete formation.

Single-cell RNA sequencing (RNA-seq) has recently been used to reveal cellular lineage changes during testicular development in gonadal sex differentiation in chicken embryos; however, there is still a lack of information on lineage changes and interactions between somatic and germ cells during post-embryonic testicular development in poultry.

Unlike mammals, whose testes descend into the scrotum facilitate thermoregulation regulation, avian species retain intra-abdominal testes. This evolutionary divergence in thermal regulation likely underpins species-specific adaptations in testicular development and spermatogenesis. Applying single-cell RNA sequencing to duck testes can reveal lineage-specific reproductive features in birds. Moreover, the reproductive success and commercial productivity of male poultry are directly influenced by testis development and semen quality. Nevertheless, the molecular and cellular regulatory mechanisms governing duck testicular development and spermatogenesis remain poorly characterized.

The study demonstrated that testicular weight in male ducks exhibited a gradual increase throughout the 24-week post-hatch period, with notable developmental differences observed across genetic lines (Hu et al., 2025). Our preliminary research on Jinding ducks revealed that testicular development significantly lags behind body weight gain. At 10 weeks of age, the ducks were approached physical maturity, however, their testes remain under developed. A marked acceleration in testicular development was observed from 16 to 22 weeks of age. Notably, approximately 40 % of the duck testes reached functional maturity and initiated sperm by 20 weeks, with complete testicular maturation and sustained sperm production by 23 weeks. Therefore, we examined duck testes at 10 and 23 weeks to determine the reproductive and somatic subpopulations at these two critical developmental stages, and compared the gene expression profiles of testicular germ cells before and after sexual maturity, to define the developmental trajectory and differences in gene expression in germ cells. Comparative transcriptomic analysis of germ cells, Sertoli cells and all testicular cells between immature (IMT) and mature (MT) testes were also conducted to identify significantly enriched pathway and genes in these cell types. The aim is to enhance our understanding of two time points of the post-embryonic development of avian testes and reveal the differences in cells before and after sexual maturity using single-cell RNA-seq. These results of this study will further our understanding of the developmental patterns in duck testes and promote research on the mechanisms of Spermatogenesis during duck testicular development.

## Materials and methods

### Experimental animals and sample collection

All experimental ducks were raised under the same feeding conditions. Five Jinding ducks were selected randomly at 10 (immature, IMT) and 23 weeks old (mature, MT), respectively, and their body weights and testicular weights were measured. One testicle was collected from each animal and placed in animal testicular tissue fixative (10 times the volume of the testicle). Paraffin sections were obtained, stained with hematoxylin and eosin, and photographed under a microscope (Eclipse Ci-L, Nikon, DJ, Japanese). The diameter of the seminiferous tubules and number of interstitial cells were measured using Image Pro Plus 6.0 analysis software (Media Cybernetics, MD, USA) and the number of stromal cells per square millimeter was calculated. The other testicle from each animal was used for single-cell sequencing. The research on live animals met the guidelines approved by the Jiangsu Institute of Poultry Science Animal Welfare and Animal Experiment Ethics Review Committee.

### Preparation of testicular tissue single-cell samples

Six testes from 10-week-old and 23-week-old ducks (three each) were washed with pre-cooled DMEM solution, excess tissue was removed, and they were cut into pieces by ophthalmology scissors, and placed in a 50 ml centrifuge tube. After brief centrifugation (300 × *g*, 1 min), the supernatant was removed and 10 mL DMEM containing mixed enzymes (including 1 % collagenase, 0.6 % DNase and 0.5 % hyaluronidase(v/v)) was added at 37°C in a water bath for about 15 minutes. Pre-cooled DMEM was then added to terminate digestion for 2 minutes, and the supernatant was removed into a 50 mL centrifuge tube using a pipette, filtered into a new 50 mL centrifuge tube using a 70 µm cell sieve, and centrifuged at 500 × *g* at 4°C for 5 minutes. The supernatant was then removed and added to the precipitate. The cells were resuspended in pre-cooled DMEM and red blood cell lysate was added to lyse red blood cells, followed by centrifugation at 400 × *g* for 5 minutes. The cells were resuspended in 100–500 µL of pre-cooled DMEM. Ten microliters of single-cell suspension were selected and the cells were stained with 0.4 % trypan blue. A cell viability ≥90 % was ensured by counting using a Countess® II Automated Cell Counter and the live-cell concentration was adjusted to the ideal concentration (1000-2000 cells/µL).

### 10 × genomics single-cell library preparation

IMT and MT testis cells were transferred to a 10 × Genomics single-cell 3′ chip according to the standard protocol. Cellular suspensions were loaded on a 10 × Genomics GemCode Single-cell instrument to generate single-cell Gel Bead-In-EMulsions (GEMs). Libraries were generated and sequenced from the cDNAs using Chromium Next GEM Single Cell 30 Reagent Kits v3.1. After dissolution of the Gel Bead in a GEM, primers containing (i) an Illumina® R1 sequence (read 1 sequencing primer), (ii) a 16 nt 10 × barcode, (iii) a 10 nt unique molecular identifier (UMI), and (iv) a poly-dT primer sequence were released and mixed with cell lysate and Master Mix. Barcoded, full-length cDNAs were then reverse-transcribed from polyadenylated mRNA. Silane magnetic beads were used to remove leftover biochemical reagents and primers from the post-GEM reaction mixture. Full-length, barcoded cDNAs were then amplified by polymerase chain reaction to generate sufficient mass for library construction. The libraries from each sample were sequenced by Gene Denovo Biotechnology Co., Ltd. (Guangzhou, China) using an Illumina NovaSeq6000 with a paired-end 150 bp reading strategy.

### Analysis of raw single-cell RNA-seq data

10 × Genomics Cell Ranger software (version 6.1.0) was used for alignment and counting. Reads with low-quality barcodes and UMIs (Unique Molecular Identifier) were filtered out and then mapped to the reference genome. Reads uniquely mapped to the transcriptome and intersecting an exon by ≥50 % were considered for UMI counting. The UMI sequences were filtered and corrected by Cell Range. Cell by gene matrices were produced via UMI counting and cell barcode calling and matrices for each sample were imported individually into Seurat version 4.1.4 for downstream analysis. The filtering thresholds were set as follows: gene counts per cell between 200 and 7 700; UMI counts per cell less than 46 000; mitochondrial gene percentage less than 20 %. Cell clusters were analyzed using the Louvain method and visualized using Uniform Manifold Approximation and Projection (UMAP) for dimension reduction analysis.

### Normalizing the data

After removing unwanted cells from the dataset, we employed a global-scaling normalization method “LogNormalize” that normalizes the gene expression measurements for each cell by the total expression, multiplies this by a scale factor (10,000 by default), and log-transforms the results. The formular is shown as follows: A gene expression level = log(1+UMI_A/_UMI_total_ × 1000).

### Cell-type annotation and marker identification

Cell-type annotation was carried out using R packages. The differentially expressed genes (DEGs) for each identified cluster were screened using the FindMarkers function with Wilcoxon’s test. A gene considered as a DEG must be expressed in 25 % of cells and have a logFoldChange (FC) ≥0.25. Cell-type-specific genes (marker genes) were identified using the criterion log2FC ≥0.36 and adjusted *p* ≤ 0.01. Heatmaps showing gene expression across cell types were created using scRNAtoolVis (https://github.com/junjunlab/ scRNAtoolVis). Cell types were identified by marker genes using CellMarker (http://bio-bigdata. hrbmu.edu.cn/CellMarker/index.jsp) (Aizarani et al., 2019) and published papers (Green et al., 2018; Xiu et al., 2021; Guo et al., 2018; Yang et al., 2021)

### Kyoto encyclopedia of genes and genomes (KEGG) analyses

DEGs in different clusters and different groups were investigated using KEGG (http://www.kegg.jp/) (Kanehisa et al., 2000). The calculated p-value was obtained by FDR correction, with FDR ≤0.05 as a threshold. Pathways meeting this condition were defined as significantly enriched pathways among the DEGs.

### Protein-protein interaction network analysis

Protein-protein interaction networks were constructed using the STRING online database (cn.string-db.org/) and Cytoscape software.

### Relative expression levels of testicular tissues by RT-PCR

qRT-PCR (quantitative reverse transcription polymerase chain reaction) was performed on 13 selected DEGs to validate the single-cell sequencing data with β-actin used as a reference gene. All primer sequences were designed using Premer Premier 5 software (Premier Biosoft, Palo Alto, CA) and are listed in Supplementary Table S10. The reactions were conducted in triplicate using SuperReal qPCR Premix (SYBR Green) master mix on an Agilent Stratagene Mx3000p qPCR system under the following amplification conditions: 95°C for 10 min, followed by 40 cycles of 95°C for 25 s, 58 °C for 25 s, 75°C for 25 s, and 72 °C for 10 min.

### Quantification and statistical analysis

Data were presented as the mean ± SD. Data were analyzed with Student’s t-test.

A P value less than 0.05 was considered to be of statistical significance.

## Results

### Comparison of testicular development in ducks at 10 and 23 weeks

**Fig. 1:**
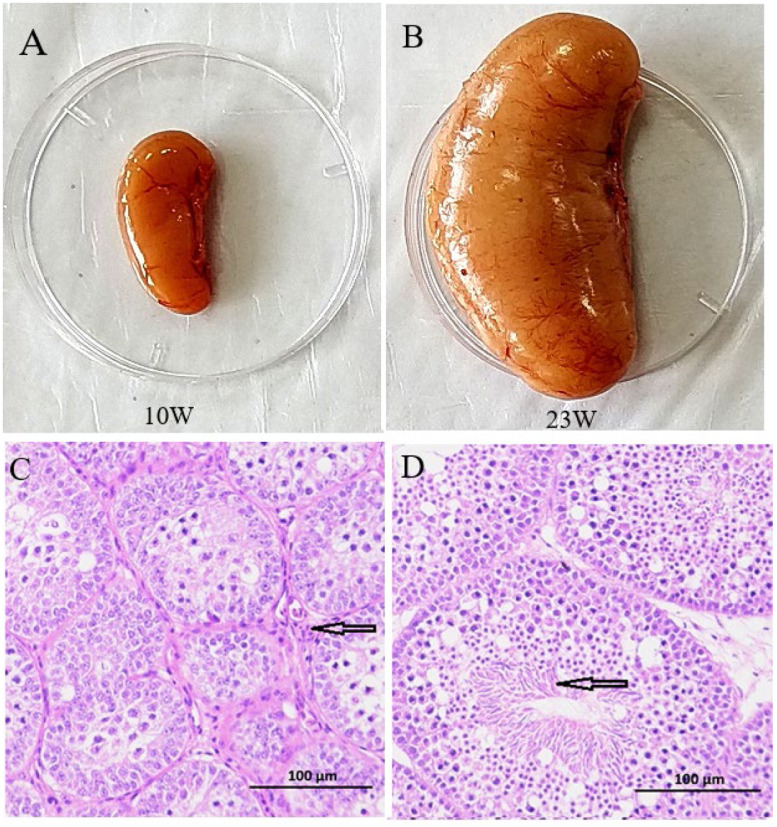
Morphological and histological comparison of testicular tissues between immature (10-week-old) and mature (23-week-old) ducks. (A) Morphology of a 10-week-old duck testis(immature). (B) Morphology of a 23 week-old duck testis(mature). (C) Histological section (H&E staining, × 200) of a 10-week-old duck testis (immature). black arrow indicates Leydig cell populations. (D) Histological section (H&E staining, × 200) of a 23-week-old duck testis (mature). (black arrow represents mature spermatozoa in seminiferous tubules.).

Body weight and testicular weight differed signifiantly between ducks at 10 and 23 weeks (Table 1), and testicular size also differed between the two periods (Fig. 1A, 1B). The area of seminiferous tubules and number of interstitial cells per unit area were both significantly larger in the testes of 23-week-old ducks compared with 10 week-old ducks (Table 1). The lumen had not yet formed at 10 weeks (Fig. 1C), but a clear lumen was visible in the seminiferous tubules at 23 weeks, and the lumen was filled with numerous mature sperm (Fig. 1D).

**Table 1:** Comparison of testicular development in IMT and MT ducks.

Group	10 weeks (IMT)	23 weeks (MT)
body weight/g	1207.222±38.864a	1451.778±106.389b
two testis total weight/g	1.471±1.427a	34.60±32.26b
number of leydig cell	658.51±258.25a	196.39±113.14b
area of seminiferous tubules (mm^2)^	13893.83±8979.74a	65728.43±19697.70bc

### RNA sequences in duck testis

More than 30 M reads were obtained in each duck testis sample. Valid barcodes over the sequence data indicated high quality, with valid barcodes >93 %, Q30 bases in barcodes >95 %, Q30 bases in RNA read >91 %, and Q30 bases in UMI >96 % (Table 2 and Supplementary Table S1). Overall, >83 % of reads were matched to the reference, with an average of 21,063–21,483 genes detected in each sample Table 2 and Supplementary Table S2). There were 7125–14,059 cells in each sample before filtering and 6567–12,446 in each sample after filtering. Finally, >1000 genes were detected per cell.

**Table 2:** Overview of duck testicular RNA sequences.

Index	IMT-1	IMT-2	IMT-3	MT-1	MT-2	MT-3
Number of Reads	332505029	340882637	336445006	327578469	343434190	300961645
Valid Barcodes (%)	94.2	93.8	94.000	97.200	97.300	96.9
Reads mapped confidently to genome (%)	83.7	84.6	83.400	83.000	83.900	84.4
Before filter Number of Cells	7125	14059	9518	11258	9864	8471
After filter Number of Cells	6567	12446	8743	10109	9010	7827
total genes detected	21063	21483	21282	21399	21475	21308
Before filter median UMI counts per cell	1543	2992	3120	6913	8360	8473
After filter median UMI counts per cell	1434	2155	2567	6750	7992	8224
Before filter median genes s per cell	1081	1737	1888	2735	3034	2892
After filter median genes per cell	1026	1379	1647	2660	2957	2797

The intragroup coefficient of variation (CV) and intergroup comparative analyses were performed on the filtered UMI counts and genes per cell above attained. The IMT group exhibited moderate variation, with CV values of 16.14 % (UMI) and 13.31 % (genes), while the MT group showed lower variability, with CVs of 5.98 % (UMI) and 3.06 %(genes), respectively. Statistically significant intergroup differences were observed with p-values of 0.001 (UMI) and 0.002 (genes)(Table 3).

**Table 3:** Analysis of intra group variation and inter group differences in UMI counts and genes per cell.

Group	Index	After filter median UMI counts per cell	After filter median genes per cell
IMT	SD	331.10	179.83
	Mean	2052.00	1350.67
	CV	16.14 %	13.31 %
MT	SD	457.59	85.82
	Mean	7655.33	2804.67
	CV	5.98 %	3.06 %
IMT vs MT	P	0.001	0.002

### Construction of single-cell atlas of duck testes

**Fig. 2:**
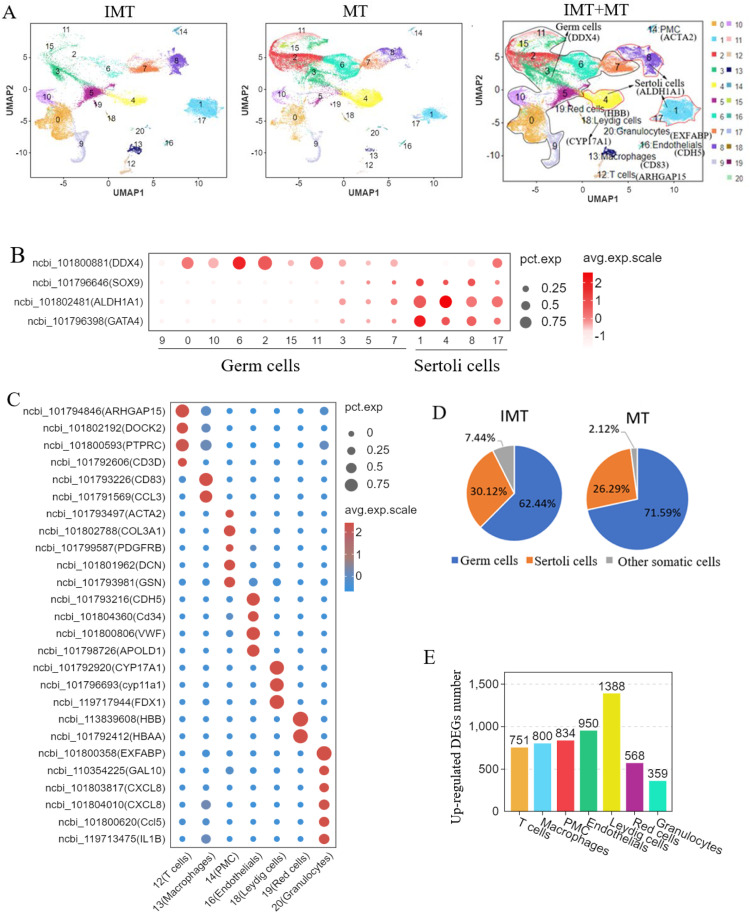
Construction of single-cell atlas of duck testes. (A) single-cell atlas of duck testes with marker gene identification. The left panel depicts the testicular cell atlas of immature ducks (IMT), the middle panel shows mature ducks (MT), and the right panel presents an integrated atlas combining both groups. Key cell populations include: Germ cells (Clusters 9, 0, 10, 6, 2, 15, 3, 5, 7), Sertoli cells (Clusters 1, 17, 4, 8), T cells (Cluster 12), Macrophages (Cluster 13), PMC (Cluster 14), Endothelial cells (Cluster 16), Leydig cells (Cluster 18), Red blood cells (Cluster 19), Granulocytes (Cluster 20). (B) Bubble chart of germ cell and Sertoli cell (SC) marker genes in duck testes. DDX4 was highly expressed in germ cell clusters (9, 0, 10, 6, 2, 15, 3, 5, 7). SOX9, ALDH1A1, and GATA4 showed high expression in Sertoli cell clusters (1, 4, 8, 17). (C) Bubble chart displaying marker gene expression profiles of testicular somatic cell populations (excluding Sertoli cells) in duck. T cells (ARHGAP15, DOCK2, PTPRC, CD3D), Macrophages (CD83, CCL3), PMC (ACTA2, COL3A1, PDGFRB, DCN, GSN), Endothelial cells (CDH5, CD34, VWF, APOLD1), Leydig cells (CYP17A1, CYP11A1, FDX1), Red blood cells (HBB, HBAA), Granulocytes (EXFABP, GAL10, CXCL8, CCL5, IL1B). (D) Comparative analysis revealed significant differences in cellular composition between IMT and MT testes. Germ cells increased from 62.44 % of IMT to 71.59 % of MT, Sertoli cell (SC) proportion decreased from30.12 % of IMT to 26.29 % of MT. (E) Bar plot showing counts of significantly upregulated genes in non-Sertoli somatic cell populations of duck testis. T cells (751), Macrophages (800), PMC (834), Endothelial cells (950), Leydig cells (1388), Red blood cells (568), Granulocytes (359).

After quality filtering, a total of 54,702 single cells were captured, including 27,756 from IMT and 26,946 from MT ducks (Supplementary Table S3). The cell cluster results were visualized in a UMAP chart (Fig. 2A). Cells were grouped into 21 clusters, including 10 germ and 11 somatic cell clusters. Somatic cell clusters included PMC (14), T cells (12), macrophages (13), endothelial cells (16), LC (18), red blood cells (19), granulocytes (20), and four SC clusters (1, 4, 8, and 17). Normalized expression of the top variable marker genes from 21 clusters are shown in a bubble chart (Fig. 2B, 2C). Clusters 0, 2, 3, 5, 6, 7, 9, 10, 11, and 15 expressed *DDX4*, which is specifically expressed in germ cells, and these 10 cell clusters were defined as germ cells. Among the somatic cells, *SOX9, GATA4*, and *ALDH1A1* were highly expressed in clusters 1, 4, 8, and 17, which were defined as SC; however, these genes were also expressed at low levels in clusters 3, 5, and 7, and *DDX4* showed high expression in cluster 7 and low expression in cluster 8 (Fig. 2B), possibly because of the close connection between germ cells and supporting cells. *ACTA2* was a marker of PMC and was specifically expressed in cluster 14, which was defined as PMC, while *COL3A1, PDGFRB, DCN*, and *GSN* were also expressed with high specificity in this cluster. Cluster 12 was defined as T cells based on *ARHGAP15, DOCK2, PTPRC*, and *CD3D* expression; cluster 13 was defined as macrophages by *CD83* and *CCL3*; cluster 16 was defined as endothelial cells by *CDH5, CD34, VWF*, and *APOLD1*; cluster 18 was defined as LC by *CYP17A1, CYP11A, FDX1*, and *STAR*; cluster 19 was defined as red blood cells by *HBB* and *HBAA*; and cluster 20 was defined as granulocytes by *EXFABP, IL1B, CCL5*, and *CXCL8* (Fig. 2C). We compared the cell ratios in the IMT and MT groups and showed a higher proportion of germ cells in MT (71.59 %) compared with IMT ducks (62.44 %), and a lower proportion of SC in MT (26.29 %) than in IMT ducks (30.12 %) (Fig. 2D). The total ratio of germ cells and SC was higher in MT (97.88 %) compared with IMT ducks (92.56 %). The detailed ratios for each cluster are shown in Supplementary Table S3. The upregulated differentially expressed genes (DEGs) across seven identified somatic cell subpopulations (excluding supporting cells) are shown in Fig. 2E. The number of upregulated DEGs varied by cell type: Leydig cells exhibited the highest count (1,388), followed by endothelial cells (950), PMCs (834), macrophages (800), T cells (751), red blood cells (568), and granulocytes (359). A comprehensive list of these genes is available in Supplementary Table S4.

### Analysis of germ cell and SC development in duck testes

**Fig. 3:**
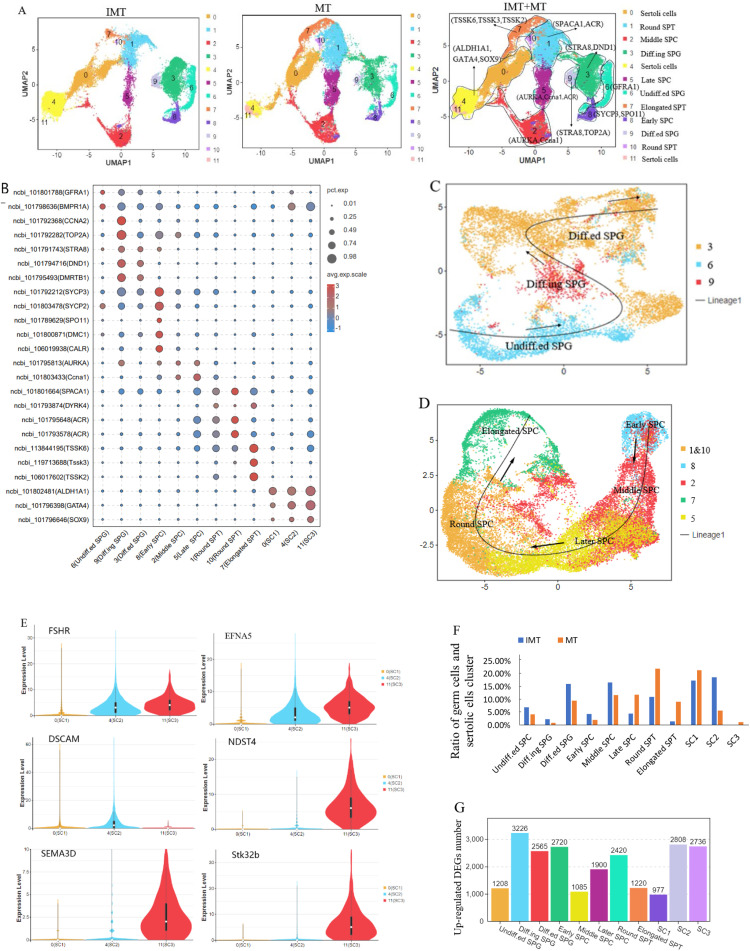
Germ cells and Sertoli cells in duck testes. (A) A single-cell atlas of duck testes identifies germ cells and Sertoli cells (SC) with their marker genes. The left panel shows the testicular cell atlas of immature (IMT) ducks, the middle panel displays mature (MT) ducks, and the right panel presents an integrated atlas of both groups. (B) Bubble chart displaying marker genes for germ cells and Sertoli cells (SC) across developmental stages in duck testes. Clusters are annotated as: Undiff. SPG (6), Diff.ing SPG (9), Diff.ed SPG (3), Early SPC (8), Middle SPC (2), Later SPC (5), Round SPT (1,10), Elongated SPT (7), and SC subtypes (SC1:0, SC2:4, SC3:11). (C) Pseudotime trajectory of duck spermatogonia (SPGs) reconstructed using Slingshot. Undiff. SPG (Cluster 6, blue), Diff.ing SPG (Cluster 9, red), and Diff.ed SPG (Cluster 3, yellow) are projected along the developmental continuum.. (D) Pseudotime trajectory of duck spermatocytes (SPCs) and spermatids (SPTs) reconstructed using Slingshot. Early SPC (cluster 8, blue), Middle SPC (cluster 2, red), Later SPC (cluster 5, yellow), Round SPT (cluster 1and10, orange), Elongated SPT(cluster 7, green). (E) Violin plots showing subtype-specific marker gene expression in Sertoli cells (SCs). *FSHR* and *EFNA5* are co-expressed in SC2 and SC3, while *DSCAM* is SC2-specific, and *NDST4, SEMA3D*, and *STK32B* are restricted to SC3. (F) Comparative analysis of cell type proportions revealed distinct distributions of germ cells and Sertoli cells (SCs) between immature (IMT) and mature (MT) duck testes. (G) Comparative transcriptomic analysis revealed the number of gene upregulation across germ cell and Sertoli cell (SC) clusters. Undiff. SPG (1208), Diff.ing SPG (3226), and Diff.ed SPG (2565,), Early SPC (2720), Middle SPC (1085), Later SPC (1900), Round SPT (2420), Elongated SPT (1220), SC1 (977), SC2 (2808), SC3 (2736).

There was overlap between germ cells and SC in the first subgrouping (Fig. 1A, 1B). To clarify the development of germ cells and SC between 10 and 23 weeks, we therefore performed a second round of cell sub-clustering of the 10 germ cell clusters (0, 2, 3, 5, 6, 7, 9, 10, 11, 15) and four SC clusters (1, 4, 8, 17) in IMT and MT ducks, including a total of 52,065 cells. This second subgrouping indicated that germ cells were divided into nine clusters (1, 2, 3, 5, 6, 7, 8, 9 and 10) and SC into three clusters (0, 4, and 11) (Fig. 3A). The nine germ cell clusters showed highly specific gene expression patterns (Fig. 3B). Cluster 6 was marked by *GFRA1*, as a marker gene of undifferentiated SPG (Wen et al., 2022), and *BMPR1A*, as a marker gene of Spermatogonial stem cells,^6^ was also highly expressed in cluster 6; cluster 6 was therefore defined as undifferentiated SPG (Undiff.ed SPG). *STRA8* and *DND1* as marker genes of spermatogonia (Li et al., 2022), and *DMRTB1* defined as a marker gene of differentiated SPG (Zhao et al., 2021), were highly expressed in clusters 9 and 3, while *CCNA2* was specifically highly expressed in cluster 9 but not in cluster 3. CCNA2 functions as a regulator of the cell cycle and binds to and activates cyclin-dependent kinases CDK1 and CDK2, thus promoting transition through G1/S and G2/M (Pagano et al., 1992). *TOP2A* also defined differentiating SPG in human testis (Guo et al., 2018). We therefore speculated that cluster 9 comprised differentiating SPG (Diff.ing SPG) and cluster 3 was differentiated SPG (Diff.ed SPG). We confirmed this speculation by developmental trajectory analysis of three subpopulations (clusters 3, 6, and 9) of spermatogonia using Slingshot software, and showed that these three clusters represented developmental changes from cluster 3 (Diff.ed SPG) through cluster 6 (Diff.ing SPG) to cluster 9 (Undiff.ed SPG) (Fig. 3C).

*SYCP3* and *SYCP2* are marker genes of early spermatocytes and were highly expressed in cluster 8, which was accordingly defined as early SPC. *SPO11, DCM1*, and *CALR* were also highly expressed in this cluster. *SPO11* is a marker gene of human leptotene SPC (Wang et al., 2018); *CALR* is defined as a marker gene of human Diff.ing SPG (Sohni et al., 2019); and *DMC1* is a marker gene of pig spermatocytes (Zhang et al., 2021). In this study, these three genes were defined as marker genes of early SPC. *Ccna1* and *AURKA* are marker gene of sheep SPC and were highly expressed in clusters 2 and 5, while *ACR* (a marker gene of later SPC) and *SPACA1* (a marker gene of round SPT) were also highly expressed in cluster 5. Cluster 5 was accordingly defined as later SPC and cluster 2 as middle SPC. *DYRK4* is a marker gene of mouse SPT (Lukassen et al., 2018a) and was highly expressed in clusters 1, 10, and 7, which were defined as SPT. Furthermore, *ACR* and *SPACA1* were highly expressed in clusters 1 and 10, and *TSSK6* was highly expressed in cluster 10. *ACR* is a marker gene of later SPC (Xiu et al., 2021), *SPACA1* is a marker gene of round SPT (Green et al., 2018), and *TSSK6* is a marker gene of elongated SPT (Grive et al., 2019). We therefore defined clusters 1 and 10 as round SPT and cluster 7 as elongated SPT. Pseudotime analysis of duck SPC and SPT confirmed the developmental trajectory from cluster 8 (early SPC) through cluster 2 (middle SPC), cluster 5 (later SPC), clusters 1 and 10 (round SPT), to cluster 7 (elongated SPT) (Fig. 3D).

Clusters 0, 4, and 11 expressed high levels of *ALDH1L1, GATA4*, and *SOX9*, which are SC marker genes, and these three clusters were therefore defined as SC (Fig. 3B). Clusters 4 and 11, but not cluster 0, also expressed high levels of *FSHR* and *EFNA5*, and *DSCAM* was relatively highly expressed in cluster 4. *NDST4, SEMA3D*, and *Stk32b* were only highly expressed in cluster 11. We therefore defined cluster 0 as SC1 with the marker genes *ALDH1L1, GATA4*, and *SOX9*, cluster 4 as SC2 with the marker genes *ALDH1L1, GATA4, SOX9, FSHR, EFNA5*, and *DSCAM*, and cluster 11 as SC3, with the marker genes *ALDH1L1, GATA4, SOX9, FSHR, EFNA5, NDST4, SEMA3D*, and *Stk32b* (Fig. 3B and 3E).

Following the second round of sub-clustering, we obtained 16754 Sertoli cells and 35311 germ cells (Table 4). The IMT group exhibited a Sertoli cell proportion of 36.11 % with corresponding germ cell constituting 63.89 %, whereas the MT group showed significantly different cellular proportions (28.35 % Sertoli cells vs 71.65 % germ cells) (Table 4). The cell ratios of eight germ cell type and three SC type in IMT and MT duck testes were detected by second subgrouping. The proportions of Undiff.ed SPG, Diff.ing SPG, Diffed.ed SPG, early SPC, and middle SPC were higher in IMT than in MT testes, but the proportions of later SPC, round SPT, and elongated SPT were lower in IMT than MT testes. Among SC, the ratio of SC1 was increased and SC2 was decreased in MT compared with IMT testes. SC3 was a new cell cluster and was only found in MT testes (Fig. 3F, Table 4). Furthermore, cell-type-specific marker genes across distinct cellular subtypes within the duck testicular cellular were cataloged in Table 5.

**Table 4:** The number and relative proportion of each germ cell and Sertoli cell cluster in IMT and MT ducks.

Cluster	Cluster name	IMT	MT	total
0	SC1	4477 (17.43 %)	5647 (21.41 %)	10124
4	SC2	4799 (18.68 %)	1513 (5.74 %)	6312
11	SC3	1 (0 %)	317 (1.2 %)	318
	total	9277(36.11 %)	7477(28.35 %)	16754
6	Undiff.ed SPG	1808 (7.04 %)	1116 (4.23 %)	2924
9	Diff.ing SPG	624 (2.43 %)	243 (0.92 %)	867
3	Diff.ed SPG	4120 (16.04 %)	2529 (9.59 %)	6649
8	Early SPC	1138 (4.43 %)	542 (2.05 %)	1680
2	Middle SPC	4300 (16.74 %)	3088 (11.71 %)	7388
5	Later SPC	1181 (4.6 %)	3126 (11.85 %)	4307
1 &10	Round SPT	2861 (11.13 %)	5832 (22.11 %)	8693
7	Elongated SPT	381 (1.48 %)	2422 (9.18 %)	2803
	total	16413(63.89 %)	18898(71.65 %)	35311
total		25690	26375	52065

**Table 5:** The list of marker gene of each testicular cell type of duck.

Cell type	Marker gene
T cell	ARHGAP15;DOCK2;PTPRC;CD3D
Macrophages	CD83;CCL3
PMC	ACTA2;COL3A1;PDGFRB;DCN;GSN
Endothelial cells	CDH5;CD34;VWF;APOLD1
Leydig cells	CYP17A1;CYP11A1;FDX1
Red cells	HBB;HBAA
Granulocytes	EXFABP;GAL10;CXCL8;CCL5;IL1B
Undiff.ed SPG	GFRA1;BMPR1A
Diff.ing SPG	CCNA2;TOP2A;STRA8;DND1;DMRTB1
Diff.ed SPG	STRA8;DND1;DMRTB1
Early SPC	SYCP3;SYCP2;SPO11;DMC1;CALR
Middle SPC	AURKA;CCNA1
Later SPC	AURKA;CCNA1;SPACA1;ACR
Round SPT	SPACA1;DYRK4;ACR
Elongated SPT	DYRK4;TSSK6;TSSK3;TSSK2
SC 1	ALDH1A1;GATA4;SOX9
SC 2	ALDH1A1;GATA4;SOX9;FSHR;ENFA5;DSCAM
SC 3	ALDH1A1;GATA4;SOX9;FSHR;ENFA5;NDST4;SEMA3D;Stk32b

The upregulated differentially expressed genes (DEGs) charactering eight identified germ subtypes and three Sertoli cell subtypes are presented in Fig. 3G. The numbers of upregulated DEGs were as follows: 1,208 in Undiff.ed SPG, 3,226 in Diffy.ing SPG, 2,565 in Diff.ed SPG, 2,720 in Early SPC, 1,085 in Middle SPC, 1,900 in Later SPC, 2,420 in Round SPT, 1,220 in Elongated SPT, 9,77 in SC1, 2,808 in SC2, and 2,736 in SC3. A detailed list of these genes is provided in Supplementary Table S5.

### Pseudo-time axis differentially analysis of germ cells

**Fig. 4:**
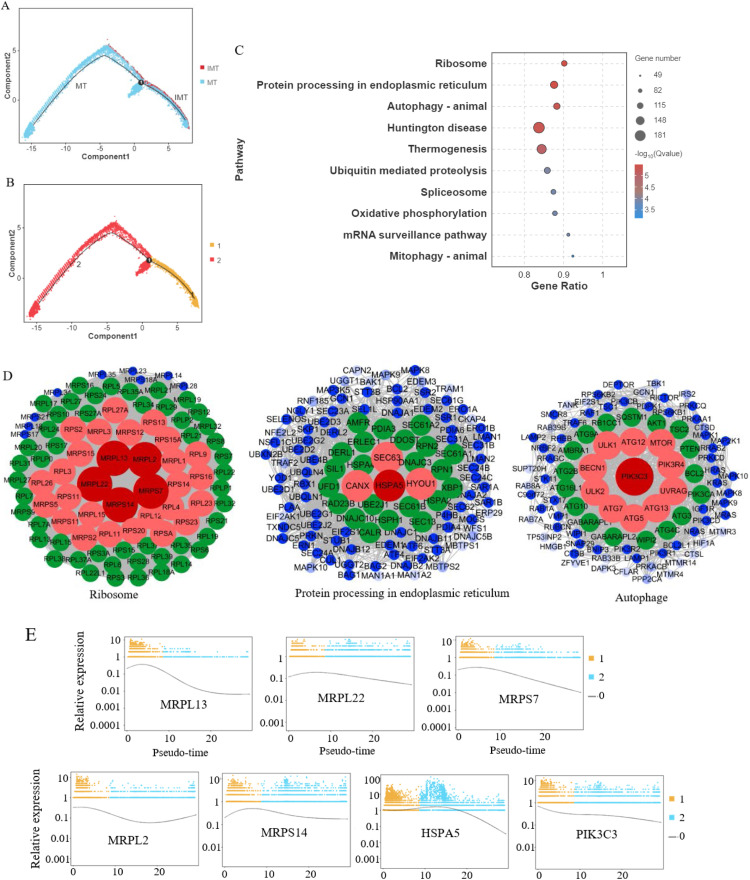
Pseudotime trajectory analysis of germ cells. (A) Pseudotime trajectory analysis of germ cells from mature (MT) and immature (IMT) duck testes, reconstructed using Monocle2. Red dots: IMT group cells; Blue dots: MT group cells; Black lines: Predicted developmental trajectories. (B) The differentiation status of MT and IMT germ cells was analyzed using Monocle2. Yellow dots represent cells in differentiation state 1, while red dots represent cells in differentiation state 2 (C) KEGG analysis of pseudotime-related differentially expressed genes. The top 10 enriched signaling pathways are shown in the figure. (D) Protein interaction network analyses were performed for the ribosome, protein processing in endoplasmic reticulum, and autophagy-animal pathway genes, respectively. In each network, the red circle at the center represents the hub genes of corresponding pathway. (E) A scatter plot showing the expression dynamics of a differentially expressed gene along the pseudotime-axis. The expression of MRPL13, MRPL22, MRPS7, MRPL2, MRPS14, HSPA5, and PIK3C3 decreased with varying magnitudes along pseudotime-axis.

Pseudotime trajectory analysis of germ cells of IMT and MT group were performed using Monocle 2. Fig. 4A displays the cellular trajectory maps of MT and IMT testicular germ cells, while Fig. 4B illustrates their differentiation status. Through this pseudotemporal ordering analysis, we identified 12,452 differentially expressed genes (Supplementary Table S6). The top 10 enriched KEGG pathways revealed significant associations with ribosome, protein processing in the endoplasmic reticulum, autophagy, Huntington's disease, thermogenesis, and related pathways.

Protein-protein interaction (PPI) network analyses of genes involved in the ribosome, protein processing in the endoplasmic reticulum and autophagy pathway are presented in Fig. 4D. Among which, MRPL13, MRPL2, MRPL22, MRPS14, and MRPS7 were identified as hub genes for the ribosome, HSPA5 as the hub gene for protein processing in the endoplasmic reticulum, and PIK3C3 as the hub gene for the autophagy. These pathway-specific hub genes demonstrated progressive downregulation along the pseudotime trajectory in germ cells (Fig. 4E).

### Difference analysis of germ cells and Sertoli cells between IMT and MT groups

**Fig. 5:**
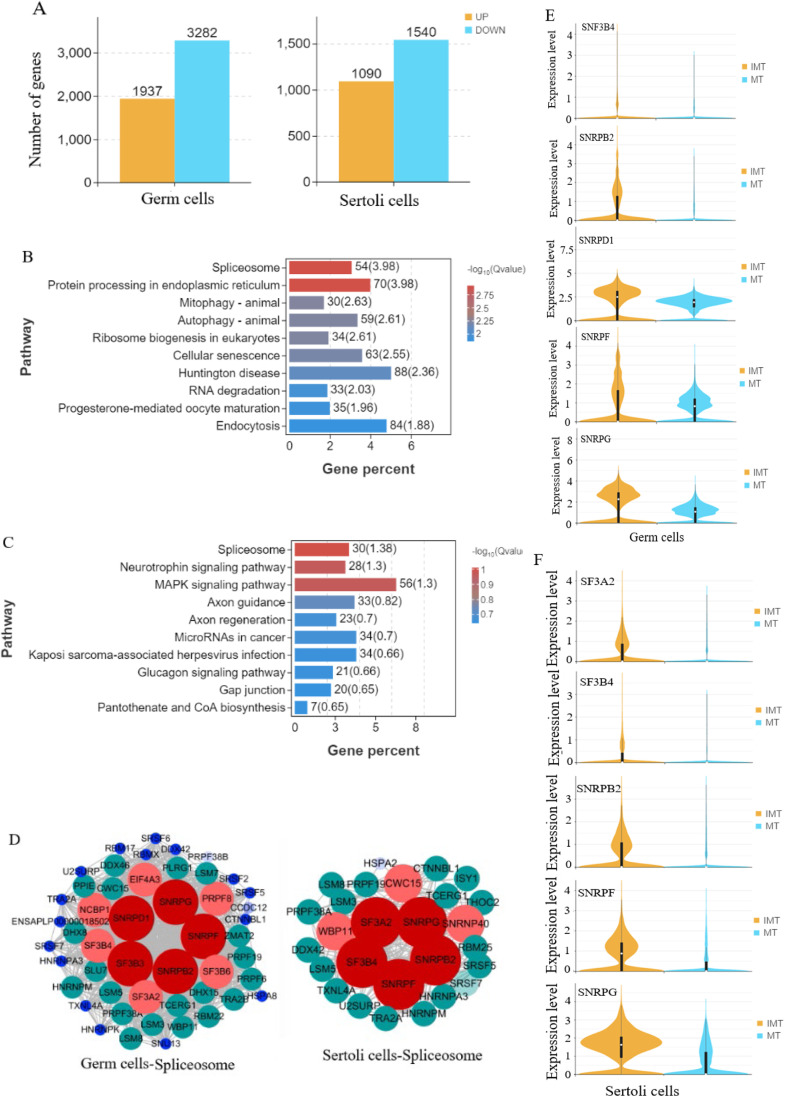
Differential analysis of germ cells and Sertoli cells between MT and IMT. (A) The number of differentially expressed genes (DEGs) in germ cells and Sertoli cells. Germ cells exhibited 1,937 upregulated and 3,282 downregulated genes, Sertoli cells showed 1,090 upregulated and 1,540 downregulated genes. (B) TOP10 KEGG pathway of germ cells between IMT and MT. The spliceosome pathway and protein processing in endoplasmic reticulum exhibited the most significant differential regulation. (C) KEGG analyses of Sertoli cells between IMT and MT. The top 10 KEGG pathways with significant differences in germ cells between IMT and MT were identified. Among these, the spliceosome pathway and protein processing in the endoplasmic reticulum pathway showed the most pronounced differential expression. (D) Protein network map of Spliceosome genes of germ cells and Sertoli cells. In germ cells, the hub genes are SNRPD1, SNRPG, SF3B3, SNRPB2, and SNRPF, whereas SF3A2, SNRPG, SF3B4, SNRPB2, and SNRPF constitute the core hubs in Sertoli cells. Notably, SNRPG, SNRPB2, and SNRPF are shared between both cell types.(E) Comparative analysis in spliceosome hub genes between IMT and MT germ cells. Compared with IMT, the expression levels of spliceosomal hub genes (SNRPD1, SNRPG, SF3B3, SNRPB2, and SNRPF) were significantly downregulated in MT germ cells. (F) Comparative analysis in spliceosome hub genes between IMT and MT Sertoli cells. Compared with IMT, the expression levels of spliceosomal hub genes (SF3A2, SNRPG, SF3B4, SNRPB2, and SNRPF) were significantly downregulated in MT Sertoli cells.

Differential expression analysis of 16,754 Sertoli cells and 35,311 germ cells between IMT and MT stages revealed 5,219 DEGs in germ cells (1,937 upregulated and 3,282 downregulated; Supplementary Table S7) and 2,630 DEGs in Sertoli cells (1,090 upregulated and 1,540 downregulated; Supplementary Table S8), as illustrated in Fig. 5A. KEGG pathway analysis revealed the spliceosome pathway as the most significantly enriched pathway in both germ cells and Sertoli cells. Notably, protein processing in the endoplasmic reticulum emerged as another highly significant pathway in germ cells (Fig. 5B), while the Neurotrophin signaling pathway and MAPK signaling pathway showed prominent enrichment in Sertoli cells (Fig. 5C).

Protein-protein interaction (PPI) network analyses of genes involved in Spliceosome pathway revealed that SNRPD1, SNRPG, SF3B3, SNRPF, and SNRPB2 were identified as hub genes for Spliceosome of germ cells, and SF3A2, SNRPG, SF3B4, SNRPB2, SNRPF were identified as hub genes for Spliceosome of Sertoli cells (Fig. 5D).

Comparative analysis of cell-type-specific Spliceosomal hubs between IMT and MT revealed that germ cell hubs (SNRPD1, SNRPG, SF3B3, SNRPB2, SNRPF) (Fig. 5E) and Sertoli cell hubs (SF3A2, SNRPG, SF3B4, SNRPB2, SNRPF) (Fig. 5F) both showed significant downregulation in the MT group.

### Difference analysis of total testis cells between IMT and MT groups

**Fig. 6:**
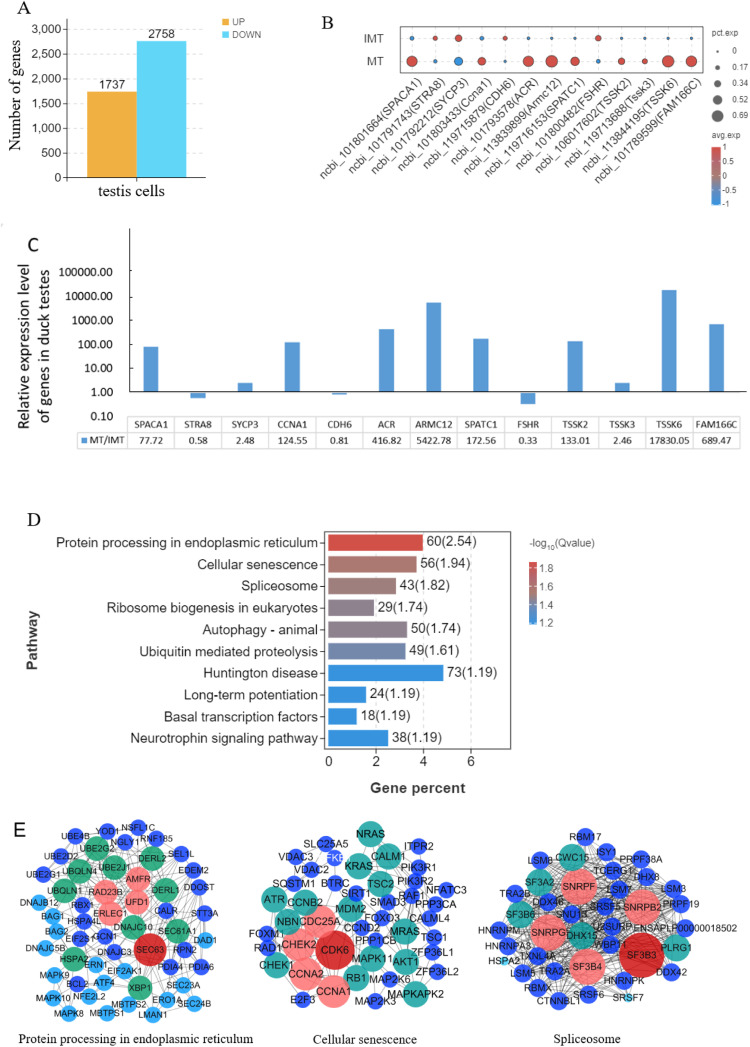
Differential analysis of total testes cells between MT and IMT. (A) Number of differentially expressed genes (DEGs) in total testes cells (4,495 total DEGs:1,737 upregulated and 2,758 downregulated). (B)Bubble chart of a part of significant DEGs of testes cells between MT and IMT detected by single cell RNA sequence. Compares to the IMT group, SPACA1, CCNA1, ACR, ARMC12, SPATC1, TSSK2, TSSK3, TSSK6, and FAM166C were upregulated in MT, while STRA8, SYCP3, CDH6, and FSHR were downregulated. (C) Relative expression levels of selected significant DEGs of duck testicular tissue between MT and IMT detected by RT-PCR. Compared with IMT, the MT group exhibited fold change of nearly 77.72 (SPACA1), 0.58 (STRA8), 2.48 (SYCP3), 124.55 (CCNA1), 0.81 (CDH6), 419.82ACR), 5422.78 (ARMC12), 172.56(SPATC1), 0.33(FSHR), 133.01 (TSSK2), 2.46 (TSSK3), 17830.05 (TSSK6), and 689.47 (FAM166C). (D) KEGG pathway analysis of total testes cells between IMT and MT. The top 10 significantly enriched KEGG pathways in total testes cells between IMT and MT were identified. Among these, the protein processing in the endoplasmic reticulum, Cellular senescence and Spliceosome pathway showed the most pronounced differential expression. (E) Protein interaction networks of genes involved in the “Protein processing in the endoplasmic reticulum”, “Cellular senescence” and “Spliceosome” pathways in total testes cells. The hub genes are identified as SEC63, CDK6, and SF3B3, respectively.

Differential expression analysis of total 54,602 testicular cells between IMT group (27,756 cells) and MT group (26,946 cells) identified 4,495 DEGs (Fig. 6A, 1737 upregulated and 2758 downregulated). A bubble chart displaying some prominent differentially expressed genes in testicular cells between MT and IMT groups revealed upregulated expression of SPACA1, CCNA1, ACR, ARMC12, SPATC1, TSSK2, TSSK3, TSSK6, and FAM166C in MT, while STRA8, SYCP3, CDH6, and FSHR showed downregulation compared to the IMT group (Fig. 6B), as detected by single-cell RNA sequencing. Subsequent RT-PCR validation in duck testicular tissues revealed distinct fold changes across target genes: SPACA1 (77.72), STRA8 (0.58), SYCP3 (2.48), CCNA1 (124.55), CDH6 (0.81), ACR (419.82), ARMC12 (5422.78), SPATC1 (172.56), FSHR (0.33), TSSK2 (133.01), TSSK3 (2.46), TSSK6 (17830.05), and FAM166C (689.47). in MT relative to IMT. KEGG pathway analysis identified protein processing in the endoplasmic reticulum, cellular senescence, and spliceosome as the most significantly enriched pathways across all testicular cells with SEC63, CDK6, and SF3B3 detected as hub genes corresponding to these pathways, respectively.

## Discussion

Here, we investigated differences between the testes in 10- and 23-week-old ducks. Testis weight was significantly lower at 10 than at 23 weeks, and the lumen had not yet formed and there were no sperm at 10 weeks, while a clear lumen filled with sperm was visible in the seminiferous tubules at 23 weeks. Ducks were therefore not sexually mature at 10 weeks and were classified as the IMT group, while the ducks had reached sexual maturity by 23 weeks and were classified as the MT group. We further investigated the differences in cell population and evolutionary relationships between the IMT and MT groups using single-cell RNA-seq technology (10 × Genomics). A total of 54,702 single cells were obtained from six duck testis (three each from IMT and MT groups) and the high-quality transcriptomes, collectively expressing >21,000 genes per sample, provided strong statistical power for unbiased analyses of these cell populations. Using a conservative statistical threshold, we identified genes that were differentially expressed between testis cells, enabling us to functionally categorize several distinct clusters. The intragroup coefficient of variation (CV) analysis of the three biological replicates in the IMT and MT groups revealed moderate variability within the IMT group and reduced variability in the MT group. Significant differences in variability were observed between the two groups, supporting the statistical validity of the experimental grouping and confirming the suitability of the dataset for downstream analyses. To the best of our knowledge, this dataset represents the first cell atlas of duck testis by single-cell transcriptomics profiling, thus providing a fundamental resource for evolutionary research.

### Annotation of somatic cell clusters with marker genes

Testicular cells include somatic cells and germ cells. Testis cell types have been studied in mammals, including mice (Green et al., 2018; Lukassen et al., 2018b; Tan et al., 2020; Zhang et al., 2023), humans (Sohni et al., 2019; Guo et al., 2018;Wang et al., 2018; Guo et al., 2020; 2021; Nie et al., 2022; Salehi et al., 2023), goats (Xiu et al., 2021; Yu et al., 2021), and yaks (Hu et al., 2022; Wang et al., 2023b). The five main somatic cell types in the testes, including PMC, endothelial cells, SC, LC, and macrophages, have been defined in each species. In addition, an innate lymphoid (type II) cell population (ILC II) and a novel mesenchymal cell population were defined in mice (Green et al., 2018), T cells were defined in goats (Xiu et al., 2021), and mast cells and T cells were defined in humans (Zhao et al., 2020). In this study, we divided somatic cells in duck testes into the following 10 types: PMC, macrophages, endothelial cells, LC, T cells, red blood cells, granulocytes, and three SC types (SC1, SC2, SC3), with each cluster distinguishable by a few marker genes. Compared with testicular cells in mammals, we identified granulocytes (cluster 20) as a new somatic cell type in ducks, with the markers *EXFABP, IL1B, CCL5*, and *CXCL8*. EXFABP is an extracellular fatty acid-binding protein expressed during chicken embryo development in hypertrophic cartilage, muscle fibers, and blood granulocyte (Descalzi Cancedda et al., 2000). Recent research showed that *EXFABP* was associated with resistance to *Salmonella* infection in chickens (Hu et al., 2022; Wang, 2023a) and may play an important role in regulating immunity and the egg-laying rate in hens (Liu et al., 2022). We found that this gene was specifically and highly expressed in cluster 20, and could thus be used to label this cluster. In addition to *EXFABP, GAL10, CXCL8, CCL5*, and *IL1B* were highly and specifically expressed in cluster 20. *GAL10* is abundantly expressed in eosinophils and basophils (Su et al., 2019), while *CXCL8* is a marker of neutrophils (Hao et al., 2021). Cluster 20 was thus defined as granulocytes, and *EXFABP* was defined as a new marker of granulocytes. However, given the small population size (117 cells), we speculated that these granulocytes may originate from peripheral blood rather than representing testicular-specific lymphocytes.

We also identified some new markers of duck testicular somatic cells. For example, *ARHGAP15* was defined as a new marker gene of T cells and *FDX1* as a new marker gene of LC. Notably, *ACTA2, PDGFRB, COL3A1, DCN*, and *GSN* were defined as markers of PMC (cluster 14). *ACTA2* and *PDGFRB* were also reported in goat and mouse testes (Green et al., 2018), and *COL3A1* and *DCN* were defined as markers of LC in chicken embryonic gonads (Estermann et al., 2020) and human testes (Sohni et al., 2019). These differences indicate the species specificity of testis cell subpopulations. Furthermore, we defined three types of SC in duck testis using the marker genes *ALDH1A1, GATA4, SOX9, FSHR, EFNA5, DSCAM, NDST4, SEMA3D*, and *Stk32b*. The ratio of SC1 was increased and SC2 was decreased during testis development from IMT to MT testes, while SC3 cells were only found in MT ducks. This suggested that different SC types may have different functions during testis development, and further studies are needed to investigate the functional differences of these three types of SC. A previous scRNA-seq study identified a second type of Sertoli cell in embryonic chicken testis, which exhibits high expression of GSTA2 and CBR4 (Estermann et al., 2020). However, in duck testis, GSTA2 is undetectable in Sertoli cells, and CBR4 shows low expression in SC1, SC2 and SC3. In this study, apart from ALDH1A1, GATA4 and SOX9 were common expression in SC1, SC2 and SC3, FSHR and EFNA5 are highly expressed in SC2 and SC3. DSCAM is specially expressed in SC2, while NDST4, SEMA3D and Stk32b are specially expressed in SC3. GATA4, SOX9, and FSHR have been reported as marker of Sertoli cells and play important roles in several species (You et al., 2021). However, the high expression of ALDH1A1, EFNA5, *DSCAM, NDST4, SEMA3D*, and *Stk32b* in Sertoli cells has not been reported previously. These differences in gene expression of Sertoli cell may reflect developmental stage variations or species-specific divergence.

### Annotation of germ cell clusters using marker genes

In mammals, there are three main categories of germ cell types: spermatogonia, spermatocytes, and spermatids; however, the reported subtypes of germ cells have differed because of different study purposes and different sample selections. For example, Nie et al. report that SPG could be further divided into undifferentiated and differentiating SPG (diff_SPG), SPC into early primary and late primary SPC, and spermatids into round and elongating/elongated spermatids (Nie et al., 2022). Wang et al. also reported that SPG were divided into SSC, differentiating SPG, and differentiated SPG, and SPC into three leptotene spermatocytes (L1, L2, L3), zygotene spermatocytes, pachytene spermatocytes, diplotene spermatocytes, and meiotic and secondary SPC, and SPT into four stages of sperm (S1–S4) (Wang et al., 2018). In the current study, we performed two subgroupings of duck testicular cells for germ cells and SC because they were not completely separated the first time and finally divided duck germ cells in the testis into eight types, including three types of spermatogonia (undifferentiated SPG, differentiating SPG, differentiated SPC), three types of spermatocytes (early SPC, middle SPC, later SPC), and two types of spermatids (round SPT, elongated SPT). These results were consistent with reports in mammals, indicating that germ cell development showed relatively conservative evolution among species.

Despite this conservative germ cell development process, however, there are significant differences in gene expression patterns, with many mammalian germ cell marker genes not found in ducks; e.g., *PIWIL4, UTF1*, and *HMGA1* are markers genes of SSC in humans (Dong et al., 2023) but were not detected in duck testis cells. In addition to the known markers detected in duck germ cell types, we also found some new marker genes, including *CCNA2* as a new marker gene of differentiating SPG in ducks. CCNA1 and CCNA2 are members of the mammalian A-type cyclin family. *CCNA1* was previously reported to be highly restricted in the testis in both mice and humans (Sweeney et al., 1996; Ravnik et al., 1996; Yang et al., 1997; Liu et al., 1998), and *CCNA1* is present in pachytene and diplotene spermatocytes but not in earlier or later stages of spermatogenesis (Panigrahi et al., 2012). *CCNA1* is also a marker of spermatocytes in sheep (Tian et al., 2022). In this text, *CCNA1* was highly expressed in middle and later SPC in ducks, but not in early SPC, coincident with the results in mice, and was accordingly defined as a marker of middle and later SPC in ducks. CCNA2 is related to the cell cycle and has been suggested as a possible molecular marker in low-grade gliomas (Qi et al., 2020), and plays a regulatory role in the development of various kinds of cancer (Zhou et al., 2024). Furthermore, the complete absence of CCNA2 leads to embryonic lethality in mice (Murphy et al., 1997). The current study provides the first report of high expression of *CCNA2* in differentiating SPG in duck testis, suggesting that it may play an important role in regulating testicular spermatogonia in ducks.

DMC1 is a recombinase that is essential for meiotic synapsis (Li et al., 2006). Mutation of *DMC1* in mice and humans often disrupts spermatogenesis and may lead to male sterility (Bannister et al., 2007; Hikiba et al., 2008). *DMC1* was recently defined as a marker of spermatocytes in pigs (Zhang et al., 2021). *DMC1* was further defined as a marker gene of early SPC in ducks, indicating a role in early-stage, but not middle- or later-stage SPC. In this study, *CALR* was also highly expressed in early SPC and can be considered as marker gene for these cells in ducks. In contrast, however, this gene is defined as a marker of differentiating SPG in humans.

*TSSK3* and *TSSK2* were also identified as new marker genes of elongated SPT in ducks. The TSSK family has six members in mice, and TSSK1-6 were all expressed post-meiotically during spermiogenesis and demonstrated an important role in germ cell differentiation and/or sperm function (Salicioni et al., 2020). *TSSK6* and double *TSSK1/TSSK2* knockout (KO) mice were sterile (Spiridonov et al., 2005; Shang et al., 2010), and sperm numbers in *TSSK3* KO mice were drastically reduced, while round spermatids were detected in the cauda epididymis, and *TSSK3* was expressed in elongating sperm and localized to the sperm tail (Nayyab et al., 2021; Nozawa et al., 2023). *TSSK6* was defined as a marker of elongated SPT in humans (Grive et al., 2019), while *TSSK5* was described as a pseudogene in humans and other primates (Zhang et al., 2010), and mouse TSSK5 might not perform as an active kinase . In this study, *TSSK1, TSSK4*, and *TSSK5* expression was absent in all stages of germ cells, and only *TSSK2, TSSK3*, and *TSSK6* were highly expressed in elongated SPT. These results suggested that *TSSK2, TSSK3*, and *TSSK6* had an important role in elongated SPT in ducks, and the functions of these three genes may differ between ducks and mammals.

We detected the ratios of the eight germ cell clusters and three SC clusters in IMT and MT testes. The results showed that the ratios changed, with a decrease in the proportion of germ cells in the early stages of development and an increase in the proportion of germ cells in the late stages of development.

Further, we identified numerous differentially expressed genes (DEGs) across distinct cell types. These findings will conductive to reveal critical insights into the roles of specific cell populations in testicular development and provide a molecular foundation to advance research on duck reproductive biology.

### Pseudo-time trajectory analysis of germ cells

The pseudo-temporal differentiation trajectory of germ cells was successfully constructed based on Monocle 2 analysis, revealing several hub genes implicated in germ cell development. Notably, mitochondrial ribosomal protein (MRP) family members including MRPL13, MRPL2, MRPL22, MRPS14, and MRPS7 were identified as critical regulators. These evolutionarily conserved genes encode essential structural and functional components of the mitochondrial complex. While previous studies have associated these MRP genes with various pathological conditions including breast cancer, diffuse large B-cell lymphoma, endometrial cancer, Leigh syndrome, and osteosarcoma (Dai et al., 2025; Zhou et al., 2025; Miao et al., 2022; Otero et al., 2025; Liu et al., 2020), and their expression has been documented in adult mouse testicular tissue . However, their specific functional roles in testicular biology remain undefined. In our analysis, these MRP genes emerged as hub components within ribosome-related pathways that were differentially regulated between IMT and MT populations. These genes exhibited consistent downregulation along the established pseudo-time axis. This coordinated expression pattern suggests these mitochondrial ribosomal components may exert negative regulatory effects on germ cell maturation and differentiation processes, potentially through modulation of ribosome biogenesis or mitochondrial translation mechanisms. The observed inverse correlation between MRP expression levels and progression along the differentiation trajectory warrants further investigation into their potential roles as developmental brakes in spermatogenesis.

HSPA5, a central mediator of the unfolded protein response (UPR), plays a critical role in maintaining cellular viability under endoplasmic reticulum (ER) stress by facilitating proper protein folding and degradation. Its essential function in supporting normal spermatogenesis and male fertility has been well-documented in murine models (Wen et al., 2023). In this study, HSPA5 emerged as a central regulatory node within the Endoplasmic Reticulum Protein Processing pathway in duck testes. This conserved functional role across species implies that HSPA5 similarly governs testicular development and spermatogenic efficiency in ducks, likely through ER stress mitigation and proteostatic regulation.

Autophagy primarily serves to maintain cellular viability under fluctuating environmental conditions (Pasquier., 2016). As a key autophagy-related gene, PIK3C3 was observed to be upregulated in Sertoli cells of non-obstructive azoospermia (NOA) patients (Hashemi Karoii et al., 2024). However, its functional role in germ cells remains undefined. This study reveals that PIK3C3 may contribute to duck germ cell development through autophagy-mediated processes. These findings advance our understanding of the molecular mechanisms governing spermatogenesis and sperm maturation.

### Difference analysis of germ cell and Sertoli cells between IMT and MT groups

The spliceosome, a highly dynamic ribonucleoprotein complex, plays critical roles in RNA processing (Wu et al., 2016). Previous studies have demonstrated that spliceosomal components, including Bud31, CWF19L2(Wang et al., 2024), and SNRPA1/U2A (Thoma et al., 2021) are functionally associated with male fertility and spermatogenesis. Our transcriptional analysis identified distinct transcriptional profiles between germ cells and Sertoli cells, with the spliceosome pathway emerging as a co-enriched pathway in both cell types. Notably, SNRPG, SF3B3 and SNRPF were identified as common hub genes of spliceosome pathway in both cellular populations, suggesting their potential role as conserved regulatory factors of cell-cell communication during testicular maturation. Mechanistically, these genes appear to exert negative regulatory effects on spermatogenesis, as evidenced by their progressive downregulation throughout testicular maturation from the immature to mature developmental stages. This discovery provides new mechanistic insights into spliceosome-mediated regulation of germ cell development and testicular maturation.

### Difference analysis of total testis cells between IMT and MT groups

Comparative analysis of total testicular cells identified differential gene expression profiles; subsequent RT-PCR validation in duck testicular tissues confirmed concordance with single-cell sequencing data, with the exception of SYCP3 exhibiting divergent expression patterns, indicating that single-cell data results are reliable. This difference may be related to differences in sample collection and processing methods. In summary, the consistency between the two methods is reached 92.3 %, suggesting results are reliable. The Protein processing in endoplasmic reticulum and Spliceosome pathway, which have been discussed as playing important roles in the development of germ cells and Sertoli cells, were identified as the prominent significantly different signaling pathways in testicular cells. These results further demonstrate their crucial involvement in testicular cell development. The cellular senescence signaling pathway emerged as another significantly enriched pathway associated with testicular development, where CDK6 (cyclin-dependent kinase 6) serves as a central regulator. As a critical component of cell cycle progression (Thoma et al., 2021), CDK6 was identified through protein-protein interaction network analysis to interact with key cell cycle regulators including CCNA2, CCNA1, CHEK2, and CDC25A, suggesting its role in orchestrating cell cycle dynamics during duck testicular development.

## Conclusions

We constructed a developmental map of duck testicular cells and identified some new marker genes, including *ARHGAP15* (T cells), *EXFABP* (granulocytes), *CCNA2* (differentiating SPGs), and *TSSK3* and *TSSK2* (elongated SPTs). Furthermore, we also the developmental changes of male germ cells in ducks from 10 to 23 weeks. Key molecular hubs—including MRPL13, MRPL2, MRPL22, MRPS14, and MRPS7 of ribosome pathway, HSPA5 of endoplasmic reticulum protein processing pathway, and PIK3C3 of autophagy pathway—were identified as molecular hubs exhibiting progressive downregulation along the differentiation trajectory. Finally, comparative transcriptomic analysis of germ cells, Sertoli cells and total testes cells between immature (IMT) and mature (MT) testes revealed that the spliceosome, protein processing in endoplasmic reticulum, and cellular senescence plays a critical regulatory role in duck testes development and spermatogenesis. The study provides data and a theoretical foundation to support further research on the function, development, and regulatory mechanisms of avian male germ cells.

## Author contributions

Data curation, Hongxiang Liu; Funding acquisition, Lizhi Lu and Huifang Li; Project administration, Wenjuan Xu; Resources, Weitao Song and Shuangjie Zhang; Writing – original draft, Zhiyun Tao; Writing – review & editing, Zhicheng Wang and Chunhong Zhu. All authors discussed and approved the manuscript.

## Funding

This research was funded by the 10.13039/501100012166National Key Research and Development Program of China (2022YFD1300100), and the JBGS Project of Seed Industry Revitalization in Jiangsu Province, China (JBGS (2021)030, JBGS (2021)111).

The authors thank The National Germplasm Center of Domestic Animal Resources for providing the experimental ducks, and Guangzhou Jidiao Biotechnology Co., Ltd. for their technical support in this experiment. The authors also thank International Science Editing (http://www.internationalscienceediting.com) for editing a draft of this manuscript.

## Lead contact

Further information and requests for resources and reagents should be directed to and will be fulfilled by the lead contact, Chunhong Zhu (zhuch_1304428@126.com) and Huifang Li (lhfxf_002@aliyun.com.cn).

### Data and code availability

The data in this study have been deposited at the GSA database of China National Center for Bioinformation (CRA020737). Raw data can be obtained through the

Shared URL: https://ngdc.cncb.ac.cn/gsa/s/x2v7aYEl.

SUPPLEMENTARY DATA

Table S1: Sequencing data for each sample;

Table S2: Comparison of results for each sample;

Table S3: Changes in proportions of single-cell subpopulations in immature and mature duck testes;

Table S4. The list of up-regulated genes of somatic cells except Sertoli cells;

Table S5. The list of upregulated genes number of germ cells and Sertoli cells;

Table S6. The list of pseudo-time differential gene;

Table S7. The list of differential genes of germ cells between IMT and MT groups;

Table S8. The list of differential genes of Sertoli cells between IMT and MT groups;

Table S9. The list of differential genes of total testicular cells between IMT and MT groups;

Table S10. The primer sequences of 13 differentially expressed genes and reference genes.

## Disclosures

The authors declare no competing interests. The funders had no role in the design of the study; in the collection, analyses, or interpretation of data; in the writing of the manuscript; or in the decision to publish the results.

## Appendix Supplementary materials

Image, application 1

Image, application 2

Image, application 3

Image, application 4

Image, application 5

Image, application 6

Image, application 7

Image, application 8
