# Supplementary material for: Construction and differential analysis of testicular atlas between 10-week-old and 23-week-old ducks using single-cell RNA sequencing
Source: Poult Sci. 2025 Aug 22;104(11):105715. doi: 10.1016/j.psj.2025.105715 (PMC12419092; doi:10.1016/j.psj.2025.105715)
Supplement: Supplementary file 1 [file mmc1.docx]

Table S1, Statistical Table of Sequencing Data for Each Sample

| Sample | Number of Reads | Valid Barcodes | | Sequencing Saturation | Q30 Bases in Barcode | Q30 Bases in RNA Read | Q30 Bases in UMI |
| --- | --- | --- | --- | --- | --- | --- | --- |
| IMT-1 | 332,505,029 | | 94.20% | 76.30% | 95.70% | 94.60% | 96.90% |
| IMT-2 | 340,882,637 | | 93.80% | 57.80% | 96.60% | 94.70% | 97.50% |
| IMT-3 | 336,445,006 | | 94.00% | 67.90% | 96.20% | 94.20% | 97.20% |
| MT-1 | 327,578,469 | | 97.20% | 44.80% | 96.60% | 91.30% | 96.20% |
| MT-2 | 343,434,190 | | 97.30% | 48.20% | 96.80% | 92.50% | 96.40% |
| MT-3 | 300,961,645 | | 96.90% | 47.40% | 96.80% | 92.10% | 96.30% |

Table S2. Statistical Table of Comparison Results for Each Sample

| Sample | Estimated Number of Cells | Fraction Reads in Cells | Mean Reads per Cell | Median Genes per Cell | Total Genes Detected | Median UMI Counts per Cell | Reads Mapped Confidently to Genome | Reads Mapped Confidently to Intergenic Regions | Reads Mapped Confidently to Intronic Regions | Reads Mapped Confidently to Exonic Regions | Reads Mapped Confidently to Transcriptome |
| --- | --- | --- | --- | --- | --- | --- | --- | --- | --- | --- | --- |
| IMT-1 | 7,125 | 68.70% | 46,667 | 1,081 | 21,063 | 1,543 | 83.70% | 9.00% | 20.60% | 54.00% | 66.70% |
| IMT-2 | 14,059 | 90.30% | 24,247 | 1,737 | 21,483 | 2,992 | 84.60% | 7.40% | 19.30% | 58.00% | 70.80% |
| IMT-3 | 9,518 | 87.00% | 35,348 | 1,888 | 21,282 | 3,120 | 83.40% | 7.40% | 16.80% | 59.20% | 69.30% |
| MT-1 | 11,258 | 84.20% | 29,097 | 2,734 | 21,399 | 6,913 | 83.00% | 8.10% | 12.70% | 62.30% | 69.50% |
| MT-2 | 9,864 | 85.40% | 34,817 | 3,034 | 21,475 | 8,360 | 83.90% | 7.60% | 13.30% | 63.00% | 71.10% |
| MT-3 | 8,471 | 85.00% | 35,528 | 2,892 | 21,308 | 8,473 | 84.40% | 8.20% | 12.20% | 64.00% | 70.90% |

Table S3. Changes in the proportion of single-cell subpopulations in immature and mature duck testes

| Cluster | Cluster name | IMT（%） | MT（%） | total |
| --- | --- | --- | --- | --- |
| 0 | Germ cell | 4878 (17.57%) | 1599 (5.93%) | 6477 |
| 2 |  | 44 (0.16%) | 5830 (21.64%) | 5874 |
| 3 |  | 2266 (8.16%) | 3199 (11.87%) | 5465 |
| 5 |  | 3652 (13.16%) | 493 (1.83%) | 4145 |
| 6 |  | 444 (1.6%) | 3582 (13.29%) | 4026 |
| 7 |  | 2765 (9.96%) | 1132 (4.2%) | 3897 |
| 9 |  | 1880 (6.77%) | 1070 (3.97%) | 2950 |
| 10 |  | 1383 (4.98%) | 549 (2.04%) | 1932 |
| 11 |  | 7 (0.03%) | 1497 (5.56%) | 1504 |
| 15 |  | 12 (0.04%) | 339 (1.26%) | 351 |
| 16 |  | 123 (0.44%) | 121 (0.45%) | 244 |
|  | total | 17454（62.44%） | 19454（71.59%） | 36865 |
| 1 | Sertoli cell | 4492 (16.18%) | 1832 (6.8%) | 6324 |
| 4 |  | 1305 (4.7%) | 3902 (14.48%) | 5207 |
| 8 |  | 2561 (9.23%) | 1157 (4.29%) | 3718 |
| 17 |  | 1 (0%) | 194 (0.72%) | 195 |
|  | total | 8359（30.12%） | 7085（26.29%） | 15444 |
| 12 | T cell | 737 (2.66%) | 58 (0.22%) | 795 |
| 13 | Macrophage | 603 (2.17%) | 78 (0.29%) | 681 |
| 14 | Pertubular myoid cells | 333 (1.2%) | 160 (0.59%) | 493 |
| 18 | Leydig cell | 98 (0.35%) | 80 (0.3%) | 178 |
| 19 | Red cell | 107 (0.39%) | 22 (0.08%) | 129 |
| 20 | Granulocytes | 65 (0.23%) | 52 (0.19%) | 117 |
| total |  | 27756 | 26946 | 54702 |
