# Supplementary material for: Construction and differential analysis of testicular atlas between 10-week-old and 23-week-old ducks using single-cell RNA sequencing
Source: Poult Sci. 2025 Aug 22;104(11):105715. doi: 10.1016/j.psj.2025.105715 (PMC12419092; doi:10.1016/j.psj.2025.105715)
Supplement: Supplementary file 8 [file mmc8.docx]

Table S10. The primer sequences of 13 differentially expressed genes and reference genes

| NCBI NO. | Gene name | Sequence（5'-3') | Sequence length |
| --- | --- | --- | --- |
| ncbi_101793578 | ACR | F: GAGGTATCAAATCAGGGCACGAC | 90 |
|  |  | R: CAGGCACGCAGGCAAGTTG |  |
| ncbi_101801664 | SPACA1 | F: CGATGGCGACTGGGACGAG | 80 |
|  |  | R: TGGACTCAAGGGCATTCAGACC |  |
| ncbi_101803433 | CCNA1 | F: GCAAACTACACGGTGAACAGGTC | 92 |
|  |  | R: TCAGTCAGGCAAGGCACTATCTC |  |
| ncbi_101792212 | SYCP3 | F: TGACACCTCCCTATGACTTTCCAG | 102 |
|  |  | R: TCTTGCCATGCTTCTCCATTACTG |  |
| ncbi_101791743 | STRA8 | F: GCCGTAGGGATGCCGAGTC | 109 |
|  |  | R: GATGCAGTGCTGTGATTGTTGAAG |  |
| ncbi_119715879 | CDH6 | F: GCGAGATAACACGGATGTCAGAG | 97 |
|  |  | R: GTAGGTTGCTAACGAGTCATACGG |  |
| ncbi_113839899 | ARMC12 | F: GTCCTCAGGCTGCTGGTCAC | 108 |
|  |  | R: CGGCTGCGAGTTGTTCAAGAG |  |
| ncbi_119716153 | SPATC1 | F: GCTGGCGGAGAACGAGGAG | 115 |
|  |  | R: GGGCGTGGAGGTGAGCAG |  |
| ncbi_113844195 | TSSK6 | F: CGGAGCTGAGCACCACCTTC | 88 |
|  |  | R: TCGTACTTCTTGGCGTCGTAGG |  |
| ncbi_106017602 | TSSK2 | F: CACTTGCTCCAGCCTGATGTATC | 95 |
|  |  | R: GGGGTTGGGATGTGGGATTTTG |  |
| ncbi_101800482 | FSHR | F: CTGAGTTGCATCGTGAGGTCTTC | 115 |
|  |  | R: GCTTGCCAGGAGAATCAGTGAG |  |
| ncbi_119713688 | TSSK3 | F: AGCTGAGCCGCACCTTCTG | 119 |
|  |  | R: AGCTGAGCCGCACCTTCTG |  |
| ncbi_101789599 | FAM166C | F: CCGTGAAGGAGATGGAGAGGTAC | 112 |
|  |  | R: GTCTGGTAGGTCCGCAGGTTC |  |
| ncbi_101800437 | β-actin | F: GCTATGTCGCCCTGGATTTC | 168 |
|  |  | R: CACAGGACTCCATACCCAAGAA |  |
